# Comparative bioactivity and immunomodulatory potential of the new Bioroot Flow and AH Plus Bioceramic sealer: An in vitro study on hPDLSCs

**DOI:** 10.1007/s00784-024-05593-7

**Published:** 2024-03-05

**Authors:** José Luis Sanz, Sergio López-García, David García-Bernal, Francisco Javier Rodríguez-Lozano, Leopoldo Forner, Adrián Lozano, Laura Murcia

**Affiliations:** 1 Departament d’Estomatologia, Facultat de Medicina I Odontologia, Universitat de València, 46010 Valencia, Spain; 2 Department of Biochemistry, Molecular Biology B and Immunology, Faculty of Medicine, University of Murcia, Biomedical Research Institute (IMIB), 30120 Murcia, Spain; 3 Department of Dermatology, Stomatology, Radiology and Physical Medicine, Morales Meseguer Hospital, Faculty of Medicine, University of Murcia, 30008 Murcia, Spain; 4 Department of Health Sciences, Catholic University San Antonio of Murcia, 30107 Murcia, Spain; 5 School of Dentistry, Hospital Morales Meseguer2 Pl.Av. Marqués de los Vélez, S/NUniversity of Murcia, 30008 Murcia, Spain

**Keywords:** Bioroot Flow, AH plus bioceramic sealer, AH plus, Bioactivity, Anti-inflammatory

## Abstract

**Objectives:**

To evaluate the cytocompatibility, bioactivity, and anti-inflammatory potential of the new pre-mixed calcium silicate cement-based sealers Bioroot Flow (BrF) and AH Plus Bioceramic Sealer (AHPbcs) on human periodontal ligament stem cells (hPDLSCs) compared to the epoxy resin-based sealer AH Plus (AHP).

**Materials and methods:**

Standardized discs and 1:1, 1:2, and 1:4 eluates of BrF, AHPbcs and AHP after setting were prepared. The following assays were performed: cell attachment and morphology via SEM, cell viability via a MTT assay, cell migration/proliferation via a wound-healing assay, cytoskeleton organization via immunofluorescence staining; cytokine release via ELISA; osteo/cemento/odontogenic marker expression via RT-qPCR, and cell mineralized nodule formation via Alizarin Red S staining. HPDLSCs were isolated from extracted third molars from healthy patients. Comparisons were made with hPDLSCs cultured in unconditioned (negative control) or osteogenic (positive control) culture media. Statistical significance was established at *p* < 0.05.

**Results:**

Both BrF and AHPbcs showed significantly positive results in the cytocompatibility assays (cell metabolic activity, migration, attachment, morphology, and cytoskeleton organization) compared with a negative control group, while AHP showed significant negative results. BrF exhibited an upregulation of at least one osteo/cementogenic marker compared to the negative and positive control groups. BrF showed a significantly higher calcified nodule formation than AHPbcs, the negative and positive control groups, while AHPbcs was higher than the negative control group. Both were also significantly higher than AHP group.

**Conclusion:**

BrF and AHPbcs exhibit adequate and comparable cytocompatibility on hPDLSCs. BrF also promoted the osteo/cementogenic differentiation of hPDLSCs. Both calcium silicate-based sealers favored the downregulation of the inflammatory cytokine IL-6 and the calcified nodule formation from hPDLSCs. BrF exerted a significantly higher influence on cell mineralization than AHPbcs.

**Clinical relevance:**

This is the first study to elucidate the biological properties and immunomodulatory potential of Bioroot Flow and AH Plus Bioceramic Sealer. The results act as supporting evidence for their use in root canal treatment.

**Supplementary information:**

The online version contains supplementary material available at 10.1007/s00784-024-05593-7.

## Introduction

Root canal treatment (RCT) comprises the chemical–mechanical disinfection of the root canal system and its subsequent filling. Root canal filling should aim to provide a three-dimensional seal of the root canal system with dimensionally stable materials in order to promote the healing of existing periapical lesions or prevent reinfection and new lesions from appearing [1].

Currently, most root canal filling techniques are based on the use of a core material i.e., gutta-percha, and an endodontic sealer. Whether cold-based techniques i.e., lateral condensation or single-cone, or techniques involving heat i.e., warm vertical compaction or continuous wave, endodontic sealers may extrude to a variable extent from the apical foramen or accessory canals and into the periodontium [2, 3]. Thus, they should at least exhibit an adequate cyto- and biocompatibility, meaning that when placed in contact with surrounding periodontal cells and tissues, respectively, no negative responses nor alterations in their physiological functioning should be expected [4].

Ideally, endodontic sealers should also exhibit bioactive properties [5, 6]. From a physical–chemical perspective, a bioactive material should be capable of inducing the precipitation of hydroxyapatite on its surface via an ionic interchange with surrounding tissue fluids. At an intra-coronal or intra-radicular level, this results in the formation of a mineral attachment to the dentin substrate [7]. From a cellular perspective, a material is considered bioactive if it influences positively on cellular plasticity. Applied to the field of Endodontics, this property is especially relevant to dental stem cells (DSCs) [8].

Within this group of mesenchymal stromal cells, human periodontal ligament stem cells (hPDLSCs) appear as potential target cells for two main reasons: 1) they are susceptible to contact with extruded intra-radicular biomaterials [9] and 2) they possess a cemento/osteo/odontogenic differentiation potential, among others [10]. This means that they can play an important role in the repair/regeneration of damaged periodontal tissue [11]. The same is the case with human stem cells from the apical papilla (hSCAPs) in immature permanent teeth [12]. For this reason, these cell lines are being used in current in vitro studies on the biological properties of endodontic cements and sealers [13–15].

Another important property of endodontic sealers is their immunomodulatory potential [16]. After the inflammation as a response to the infection resolves, local DSCs can migrate, proliferate, and differentiate to promote tissue neoformation [17, 18]. The inflammatory response towards endodontic sealers has been assessed by recent studies [19].﻿ Specifically, several studies described the potential immunomodulatory impact of endodontic sealers in terms of macrophage polarization and inflammatory cytokine production, which could promote healing, tissue repair, and inhibit inflammation [20].

Among endodontic biomaterials, calcium silicate-based sealers (CSSs) and cements (CSCs) have recently gained relevancy among the scientific community [21, 22]. This subgroup of dental materials present variable proportions of calcium and silicates in their composition and release calcium hydroxide as a subproduct of their hydraulic setting [23]. As a result, they exhibit an adequate cytocompatibility and bioactive properties. Nonetheless, variations in their composition may result in differences in their characteristics [24]. Thus, the comparison of the biological properties of new CSS compositions with established CSSs on different dental cell lineages is commonly assessed [25–27].

Most recently, the new CSSs Bioroot Flow (Septodont, Saint-Maur-Des-Fossés, France) has been introduced. According to its manufacturer, this pre-mixed tricalcium silicate-based sealer presents both biocompatibility and bioactive properties. Nevertheless, to the authors’ knowledge the biological and immunomodulatory properties of this CSS have not been assessed nor compared with other pre-mixed CSSs or established endodontic sealers.

Accordingly, the aim of the present in vitro cellular study is to compare the cytocompatibility, bioactivity and immunomodulatory potential of Bioroot Flow with the CSS AH Plus Bioceramic Sealer (Maruchi, Taejanggongdan-gil, Wonju-si, Gangwon-do, Korea) and the epoxy resin-based sealer AH Plus (Dentsply DeTrey GmbH, Konstanz, Germany) on hPDLSCs.

## Materials and methods

The manuscript of this laboratory study has been formatted in accordance with the “Preferred Reporting Items for Laboratory studies in Endodontology (PRILE) 2021” guidelines [28]. The PRILE 2021 checklist of items is presented in Supplementary Table 1.

### Sample preparation: material discs and extraction media

The composition, manufacturer, and batch number of the tested endodontic sealers (Bioroot Flow (BrF), AH Plus Bioceramic Sealer (AHPbcs) and AH Plus (AHP) are presented in Table 1.

**Table 1:** Data on the tested materials

Material	Manufacturer	Composition*	Batch Number
Bioroot Flow	Septodont, Saint-Maur-des-Fossés; France	tricalcium silicate, propylene glycol, povidone, calcium carbonate, aerosil, zirconium oxide, acrylamide / sodium acryloyldimethyltaurate copolymer, isohexadecane, polysorbate	B29728A
AH Plus Bioceramic Sealer	Manufactured by Maruchi, Taejanggongdan-gil, Wonju-si, Gangwon-do, Korea Distributed by Dentsply DeTrey GmbH, Konstanz, Germany	Zirconium dioxide (50–75%), tricalcium silicate (5–15%), dimethyl sulfoxide (10–30%), lithium carbonate (< 0.5%), thickening agent (< 6%)	KI221111
AH Plus	Dentsply DeTrey GmbH, Konstanz, Germany	Paste A: bisphenol-A epoxy resin, bisphenol-F epoxy resin, calcium tungstate, zirconium oxide, silica, iron oxide pigments Paste B: dibenzyldiamine, aminoadamantane, tricyclodecane-diamine, calcium tungstate, zirconium oxide, silica, silicone oil	2,211,000,712

^*^The percentage by weight (WT%) of each component of the tested materials is reported within brackets, if available. Composition data was extracted from the respective Material Safety Data Sheets

Fiteen discs of the tested sealers were prepared (*n* = 5 for each group). The sealers were placed into cylindrical rubber molds with standardized dimensions (diameter: 5 mm, height: 2 mm) with Hank’s balanced salt solution (HBSS; H6648; Sigma Aldrich, Gillingham, UK). Molds were previously sterilized under ultraviolet radiation for 15 min. Samples were then left to set for 48 h in an incubator (37ºC, 5% CO2, and 95% humidity). The tested sealers were handled following their respective manufacturers’ instructions: BrF and AHPbcs were placed directly to the rubber molds from their injectable pre-mixed syringes, while AHP’s double paste format was previously mixed before its placement.

For the cellular assays, samples were prepared in accordance with the International Standard ISO 10993–5 guidelines with regards to the tests for in vitro cytotoxicity and 10,993–12 for sample preparation and reference materials. Firstly, sample extracts/eluates were obtained from the tested sealers under sterile conditions. The extraction vehicle used was Dulbecco’s Modified Eagle Medium (DMEM; Gibco, Invitrogen, Waltham, MA, USA) with 10% of foetal bovine serum (FBS). Samples were immersed in DMEM for 24 h in a humid atmosphere (37ºC, 5% CO_2_) in a ratio of 3 cm^2^ of sample surface per milliliter of volume of medium and submitted to continuous agitation. Finally, three dilutions of the extraction medium were prepared using fresh DMEM (1:1, 1:2, and 1:4 v/v), based on a previous similar study [29].

### Sample preparation: isolation, culture, and characterization of hPDLSCs

The cellular extraction protocol was approved by the Human Research Ethics Committee from *Universidad de Murcia* (ID: 3686/2021) HPDLSCs were isolated from third molars from 18–30-year-old healthy patients (*n* = 10), extracted for orthodontic or periodontal reasons with a previous written informed consent. The sample size was based on a previous study on hPDLSCs [30].

Molars extracted from the subjects were immersed in Minimum Essential Medium with Alpha modifications (α-MEM; Gibco, USA) containing 1% penicillin/streptomycin (Sigma Aldrich, St. Louis, MO, USA) and amphotericin B (Fungizone; Sigma Aldrich, USA), and preserved at 4ºC. Subsequently, the teeth underwent a thorough rinsing with phosphate-buffered saline (PBS) (Gibco, USA), followed by the removal of periodontal tissues from the middle and apical thirds of their roots. These excised periodontal tissues were then fragmented into smaller pieces and subjected to digestion using Collagenase type I solution (3 mg/mL; Gibco, USA) for one hour at 37 ºC. The resulting periodontal cells were cultured in α-MEM supplemented with 10% foetal bovine serum (FBS; Sigma Aldrich, USA) and 1% penicillin/streptomycin (Sigma Aldrich, USA).

Prior to their application in the in vitro biological experiments, characterization of hPDLSCs adhered to the International Society of Cellular Therapy (ISCT) guidelines [31] was conducted to affirm their mesenchymal characteristics. The procedure entailed subjecting the cells to flow cytometry analysis (FACSCalibur Flow Cytometry System; BD Biosciences, San José, CA, USA). In brief, 1 × 10^5^ cells were resuspended in 100 mL of phosphate buffer saline (PBS) with 1% FBS and the following fluorescence-conjugated specific monoclonal antibodies: CD14, CD20, CD34, CD45, CD73, CD90, and CD105 (Miltenyi Biotec, Bergish Gladbach, Germany). The characterization process was performed using the methodology of previous similar studies [32, 33].

In addition, the characterized hPDLSCs underwent cultivation in diverse media (osteogenic/adipogenic/chondrogenic) (Miltenyi Biotec, Bergisch Gladbach, Germany) to validate their trilineage mesenchymal differentiation potential. The mesenchymal nature and trilineage differentiation ability of the hPDLSCs used in this study were corroborated by a previous investigation carried out by our research group [34]. Subsequent in vitro experiments utilized cells from passages 2–4, consistent with methodologies applied in comparable studies [35, 36].

### MTT assay: sealer cytotoxicity analysis

The cytotoxicity assessment of the three eluates (1:1, 1:2, and 1:4) derived from BrF, AHPbcs, and AHP cultured with hPDLSCs (test groups) was conducted and compared with hPDLSCs cultured in unconditioned growth medium (negative control group). The evaluation utilized a 3-(4,5-dimethylthiazol-2-yl)-2,5-diphenyltetrazolium bromide (MTT) assay, as reported in previous studies [37, 38]. Briefly, hPDLSCs were seeded onto 96-well plates with 180 μL of DMEM and incubated for 24 h at 37 °C, 5% CO2, and 95% humidity. The material eluates were introduced into the culture medium with 1 × 10^4^ hPDLSCs (*n* = 3 per test group). MTT reagent (Sigma Aldrich, USA) was applied for 4 h according to the manufacturer’s instructions. Upon detection of a purple precipitate, dimethylsulfoxide (DMSO; Sigma-Aldrich, USA) was added to each well (100 μl/well). Plates were covered and kept in dark conditions for 4 h to solubilize the formazan crystals produced by metabolically active/viable cells, post-reduction of the MTT reagent. Absorbance per well at 570 nm wavelength was recorded using a microplate reader (ELx800; Bio-Tek Instruments, Winooski, VT, United States) after 24, 48, and 72 h of culture. Replacements of the culture media with fresh eluates from the tested groups were performed every three days.

### Horizontal wound healing assay: cell migration/proliferation assessment

Migration and proliferation of hPDLSCs were evaluated via a wound healing assay, as performed in previous similar studies [37, 39], following culture in growth medium with eluates (1:1, 1:2, and 1:4) derived from BrF, AHPbcs, and AHP. A comparison was made with cells cultured in unconditioned growth medium (negative control group). HDPLSCs were seeded onto 6-well plates (2 × 10^5^ cells per well; *n* = 3 for each experimental condition) and allowed to proliferate until cell confluency was achieved. Subsequently, a superficial scratch wound was generated on each cell monolayer using a 200-μL sterilized pipette tip, and each well underwent three rinses to eliminate any remaining cell debris.

At each time point, the percentage of open wound area was quantified for each sample using Image J software (National Institutes of Health, Bethesda, MD, USA) at three time points (24, 28, and 72 h of culture). Migration rates were expressed as the percentage areas of relative wound closure (RWC), accounting for width variations among the scratch wounds. RWC values were calculated as follows: RWC = (wound closure area (in pixels) / total number of pixels) × 100. Results were presented as the percentage of the total wound area at the three time points relative to the total wound area at 0 h for each respective well. Wound closure/healing was assessed for all experimental conditions in triplicate (test groups and negative control).

### Immunofluorescence: hPDLSC F-actin cytoskeleton staining

To assess variations in the morphology, structure, and organization of the F-actin cytoskeleton of hPDLSCs under exposure to the different sealer eluates, a qualitative description of immunofluorescence images of phalloidin-stained cells was performed. To do so, hPDLSCs were seeded onto glass coverslips, left to adhere, and cultured in DMEM (control) or in DMEM treated with 1:1, 1:2, or 1:4 of BrF, AHPbcs, or AHP for 72 h at 37ºC. Then, the following was performed: 1) cells were rinsed twice using pre-warmed foetal bovine serum at 37ºC; 2) cells were fixed in 4% formaldehyde solution (Merck Millipore, Darmstadt, Germany) for 10 min; 3) cells were made permeable with 0.25% Triton X-100 solution (Sigma-Aldrich) for 5 min; and 4) cell cytoskeleton and nuclei were stained with AlexaFluor™594-conjugated phalloidin (Invitrogen) and 4,6-diamidino-2-phenylindole dihydrochloride (DAPI) (ThermoFisher Scientific, Waltham, MA, United States), respectively. Lastly, immunofluorescence images were obtained and observed under a confocal microscope (Leica TCS SP2; Leica, Wetzlar, Germany). Each experimental condition and visualization were performed in triplicate.

### SEM: Cell morphology and attachment visualization

The fifteen standardized sealer sample discs prepared with the previously mentioned methodology (*n* = 5 per sealer) were selected for SEM, to assess hPDLSC morphology and attachment to the material samples, based on a previous cellular study on dental materials [40]. To do so, the discs’ surfaces were seeded with 5 × 10^4^ hPDLSCs and cultured in normal growth medium for 72 h. Following this incubation period, cells were fixed with 4% glutaraldehyde (Sigma-Aldrich, USA) in PBS for 4 h. Subsequently, the cells underwent dehydration through a series of gradually increasing ethanol dilutions (30 to 90% v/v) and were treated with hexamethyldisilazane (Sigma-Aldrich, USA) for 5 min. Finally, the cells were air-dried, sputter-coated with gold and palladium, and subjected to examination using a SEM unit (Jeol 6100 EDAX; Jeol Inc., Peabody, MA, USA). 100x, 300x, and 1500 × magnifications were used.

### RT-qPCR assay: Cell differentiation marker expression

The expression of osteo/cemento/odontogenic markers by hPDLSCs co-cultured with the materials was evaluated through real-time quantitative polymerase chain reaction (RT-qPCR). This assay acts as an indicator of cell differentiation and has also been used in previous similar studies [33, 41]. AHP was excluded from the marker expression assay because of its negative results in the hPDLSC cytocompatibility assays.

Twenty thousand hPDLSCs per well were seeded onto 12-well plates (*n* = 3) and incubated for 7 and 21 days with undiluted (1:1) sealer-conditioned medium from BrF and AHPbcs. HPDLSCs cultured in unconditioned medium acted as the negative control group, and cells cultured in osteogenic differentiation medium (OsteoDiff media; Miltenyi Biotec, Germany) served as the positive control group. Approximately 80% cell confluency was obtained at the start of the treatment with the tested sealers. 100% cell confluency was reached after 3 days of culture. The culture media with fresh eluates from the respective groups were renewed every three days. The preparation of the sealer-conditioned medium involved immersing the previously conditioned standardized sealer discs in culture medium (DMEM; Gibco, USA) for 24 h.

Total RNA extraction from hPDLSCs was carried out using the Rneasy Mini Kit (Qiagen, Hilden, Germany). Subsequently, 1 μg of RNA underwent reverse transcription for first-strand complementary DNA (cDNA) synthesis using iScript™ Reverse Transcription Supermix for RT-qPCR (Bio-Rad Laboratories Inc., Hercules, CA, USA). Both procedures adhered to the instructions provided by their respective kit manufacturers.

The primer sequences for the differentiation markers utilized in the assay were as follows (5’-3’): Cementum attachment protein or CAP (forward: TTTTTCTGGTCGCGTGGACT, reverse: TCACCAGCAACTCCAACAGG), cementum protein 1 or CEMP1 (forward: GGGCACATCAAGCACTGACAG, reverse: CCCTTAGGAAGTGGCTGTCCAG), runt-related transcription factor 2 or RUNX2 (forward: TCCACACCATTAGGGACCATC, reverse: TGCTAATGCTTCGTGTTTCCA), bone sialoprotein or BSP (forward: TGCCTTGAGCCTGCTTCCT, reverse: CTGAGCAAAATTAAAGCAGTCTTCA),

The expression of differentiation markers was quantified relative to the housekeeping gene Glyceraldehyde 3-phosphate dehydrogenase (GAPDH), with the following sequence (5’-3’): (forward: TCAGCAATGCCTCCTGCAC, reverse: TCTGGGTGGCAGTGATGG). The calculation of relative gene expression utilized the standardized 2‑ΔΔCT method [42].

### Alizarin Red S staining:cell mineralization analysis via calcified nodule formation

Alizarin Red S staining (ARS) was conducted to evaluate hPDLSC calcified nodule formation in contact with the tested sealers (BrF, AHPbcs, and AHP) to measure of their biomineralization ability, as performed in similar studies [43, 44]. Twenty thousand hPDLSCs per well were seeded onto 12-well plates (*n* = 3) and allowed to proliferate until confluency was attained.

For this assay, both a negative control (hPDLSCs cultured in unconditioned growth medium (DMEM; Gibco, USA)) and a positive control (hPLDSCs cultured in osteogenic medium (OsteoDiff; Miltenyi Biotec, Germany) were included for reference.

The cells were transferred into undiluted (1:1) sealer-conditioned medium and cultured for 21 days. Following the culture period, the samples were rinsed with foetal bovine serum and fixed with 70% ethanol for 1 h. The fixed samples were then stained with a 2% Alizarin Red solution (Sigma Aldrich, USA) for 30 min under controlled conditions (dark ambient and room temperature) and solubilized using a 10% cetylpyridinium chloride monohydrate solution (Sigma-Aldrich, USA). Finally, a Synergy H1 multi-mode microplate reader (BioTek, Winooski, VT, USA) was used to measure the absorbance values of the samples at 405 nm.

### ELISA: interleukin expression analysis

The expression of IL-6 and IL-8 from hPDLSCs was assessed by means of enzyme-linked immunosorbent assay or ELISA (FineTest ELISA kit, FineTest Biotech Inc., Boulder, CO, USA), based on a previous study on hPDLSCs [32]. To do so, hDPLSCs were seeded onto 24-well plates (5 × 10^4^ cells per well; *n* = 3 for each experimental condition) and allowed to adhere for 24 h at 37 °C, 5% CO2, and 95% humidity. Then, BrF, AHPbcs, or AHP were added into the wells and incubated for 72 h. After the incubation period, the supernatants were collected and centrifuged at 2500 rpm at 2–8 °C for 5 min to remove the cells. The ELISA was performed following its manufacturer’s instructions and the absorbance per well at 450 nm wavelength was recorded using a microplate reader (Elx800; Bio-Tek Instruments, Winooski, VT, United States).

### Statistical analysis

All the experimental conditions and measurements were performed in triplicate for each of the tested sealers (BrF, AHPbcs, and AHP). Data were expressed as mean ± standard deviations (SD). A Q-Q plot was previously performed to confirm the normality in the distribution of the data. The statistical analysis was performed using one or two-way ANOVA and Tukey’s post hoc test using Graph-Pad Prism v8.1.0 (GraphPad Software, San Diego, CA, USA). To perform the one-way ANOVA test, data was grouped by time (24 h, 48 h, and 72 h) and analysed independently. Each dilution/eluate was considered an independent experimental condition. Statistical significance was set at *p* < 0.05.

## Results

### hPDLSC characterization (flow cytometry)

**Fig. 1:**
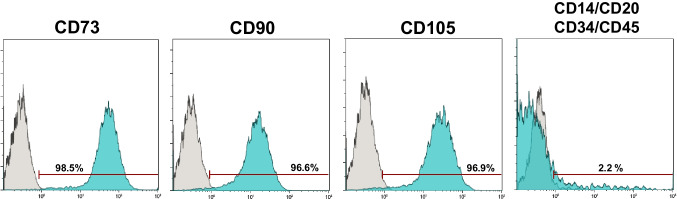
Results from the SEM–EDS analysis for the tested sealers (BrF (row A), AHPbcs (row B), AHP (row C)). The first column illustrates SEM images of each sealer (scale bar: 50 μm). The second column shows each EDS elemental spectrum. The third column lists the elements present per sealer by weight and atomic weight

The results of the flow cytometry assay for hPDLSC characterization are shown in Fig. 1. A high expression of mesenchymal stem cell (MSC)-specific surface markers CD73, CD90, and CD105, together with a low expression of hematopoietic markers CD34, CD45, CD14, and CD20 was observed. Thus, the mesenchymal phenotype of the cellular sample was confirmed.

### Sealer cytocompatibility (MTT, wound healing, immunofluorescence, and SEM assays)

**Fig. 2:**
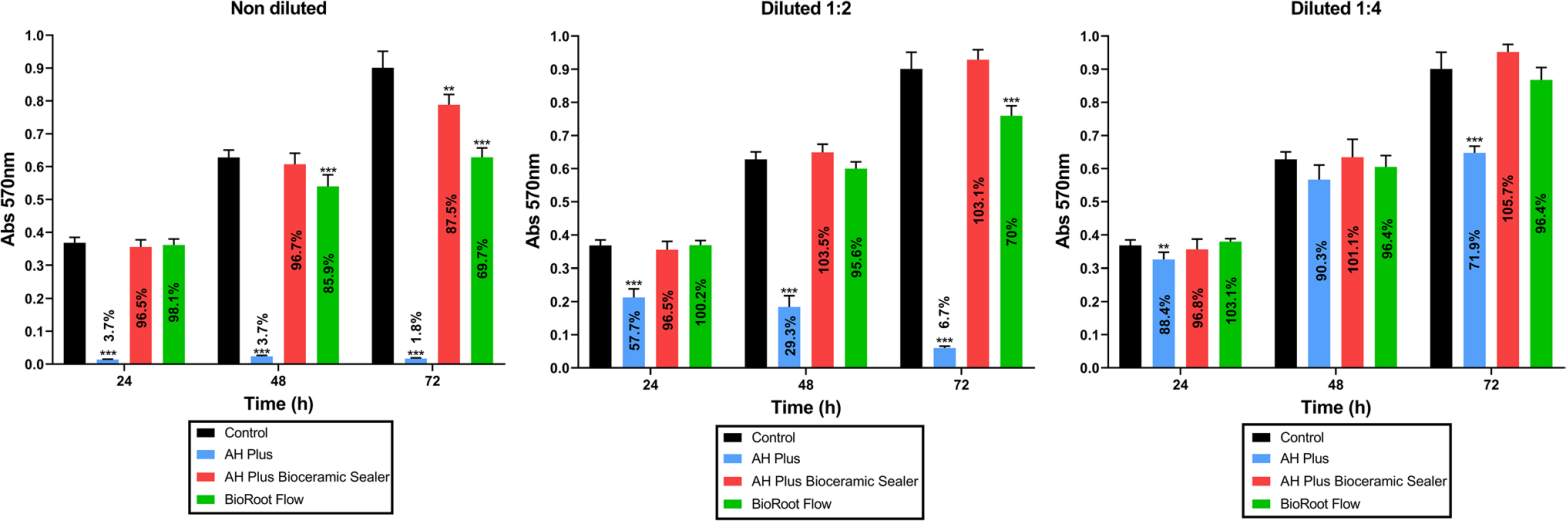
Results from the MTT assay for the 1:1, 1:2 and 1:4 eluates of the tested sealers (Brf, AHPbcs, and AHP) cultured with hPDLSCs (time points: after 24, 48, and 72 h). Data are presented absorbance values (570 nm) compared to the negative control group. *p < 0.05; **p < 0.01; ***p < 0.001 (One-way ANOVA analysis). The percentages of viable cells, calculated with the formula: (%) = [100 × (sample abs) / (control abs)], are presented in each bar

The results of the MTT assay performed to quantify hPDLSC metabolic activity and assess sealer cytotoxicity are shown in Fig. 2. At all measurement time points and dilutions, AHP-treated cells exhibited a significantly lower metabolic activity compared to the control group (*p* < 0.001). On the other hand, 1:2 and 1:4 AHPbcs, and 1:4 BrF-treated cells exhibited non-significant differences with the control group at every measurement time point (*p* > 0.05).

**Fig. 3:**
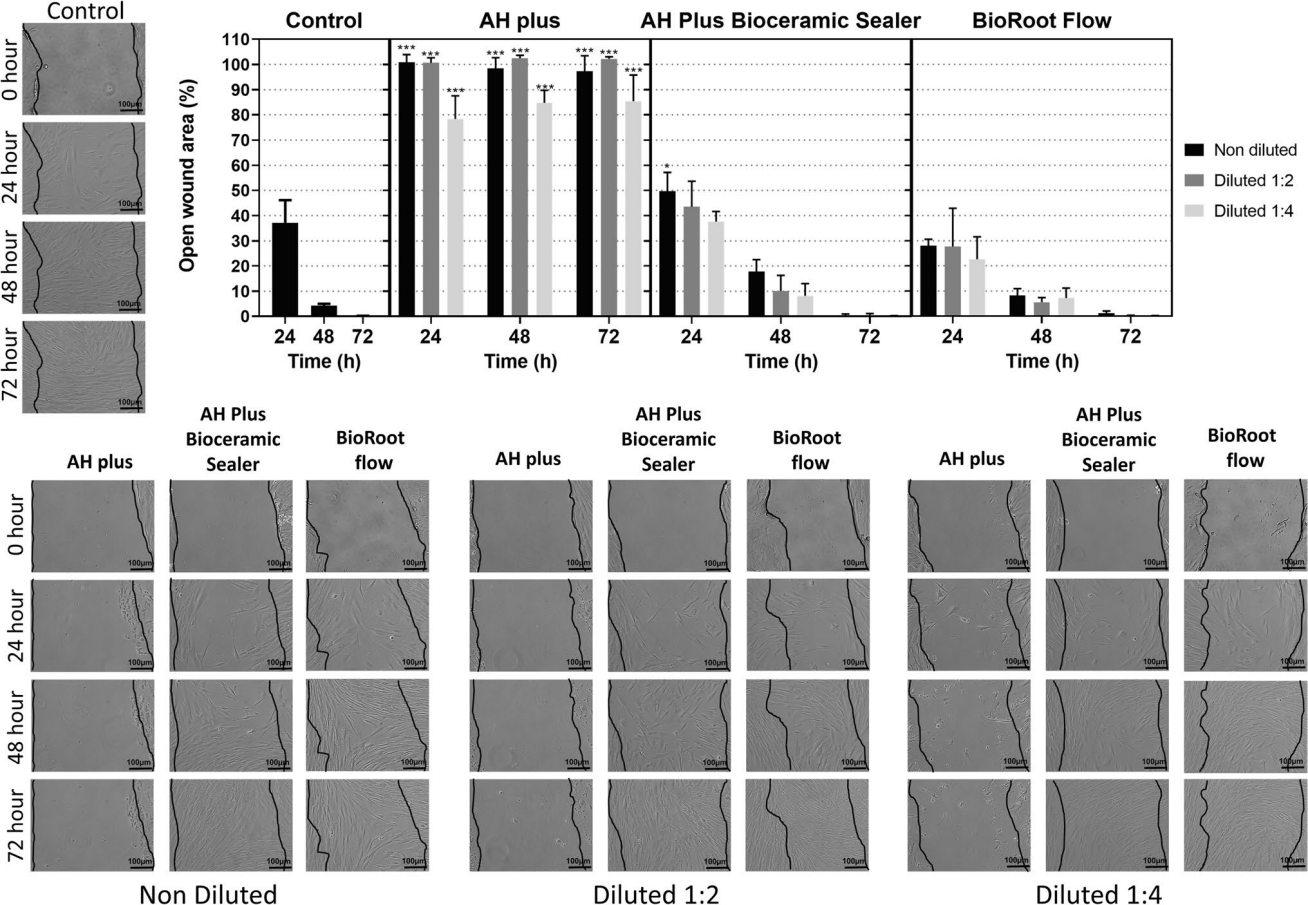
Results from the wound healing assay for the 1:1, 1:2 and 1:4 eluates of the tested sealers (BrF, AHPbcs, and AHP) cultured with hPDLSCs (time points: after 24, 48, and 72 h). Graphical results are presented as percentages of open wound areas compared to the negative control group. ***p < 0.001(One-way ANOVA analysis). Scale bar for the images: 100 µm

The results and representative images of the horizontal wound healing assay performed to quantify hPDLSC migration and assess sealer cytocompatibility are depicted in Fig. 3. At all measurement time points and dilutions, AHP-treated cells exhibited a significantly lower migration (higher percentage of open wound area) compared to the control group (*p* < 0.001). On the other hand, 1:2 and 1:4 AHPbcs, and BrF-treated cells exhibited non-significant differences with the control group at every measurement time point (*p* > 0.05).

**Fig. 4:**
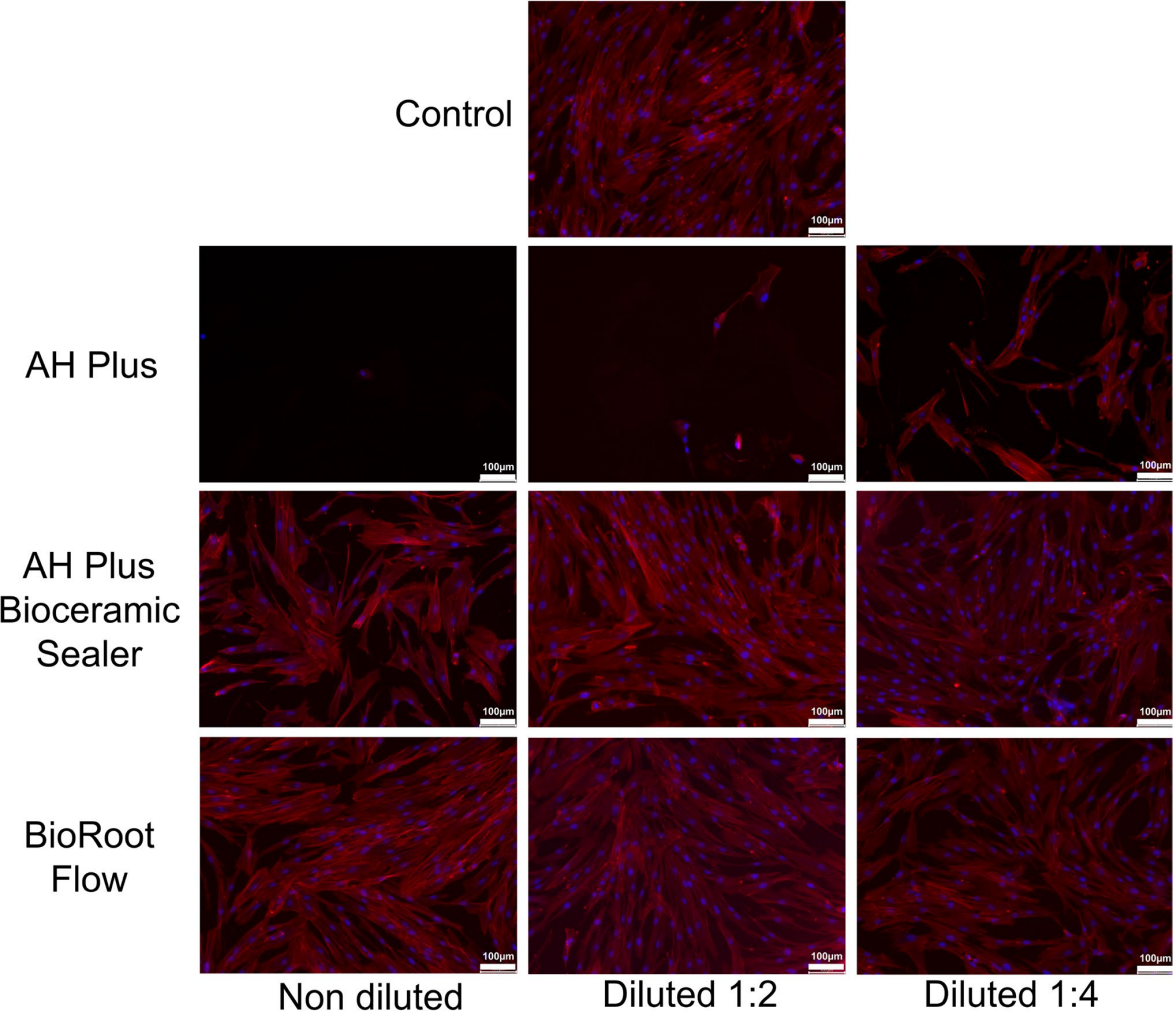
Results from the hPDLSC cytoskeleton staining after 72 h of culture with the undiluted testes sealers (BrF, AHPbs, and AHP). Scale bar: 100 µm

Representative results of the immunofluorescence staining performed to qualitatively assess variations in the morphology, structure, and organization of the F-actin cytoskeleton of hPDLSCs treated with the tested sealers are illustrated in Fig. 4. AHP-treated groups exhibited a small number of cells with an aberrant morphology at 1:1 and 1:2 dilutions, and a low count of spindle-like cells at 1:4 dilution. Contrarily, all dilutions of BrF and AHPbcs-treated groups evidenced a wide spread of hPDLSCs fibroblast-like spindle-shaped morphology and a high F-actin content, comparable to that of the control group. Furthermore, at 1:4 dilution, a higher number of functionally oriented cells were observed in both cases.

**Fig. 5:**
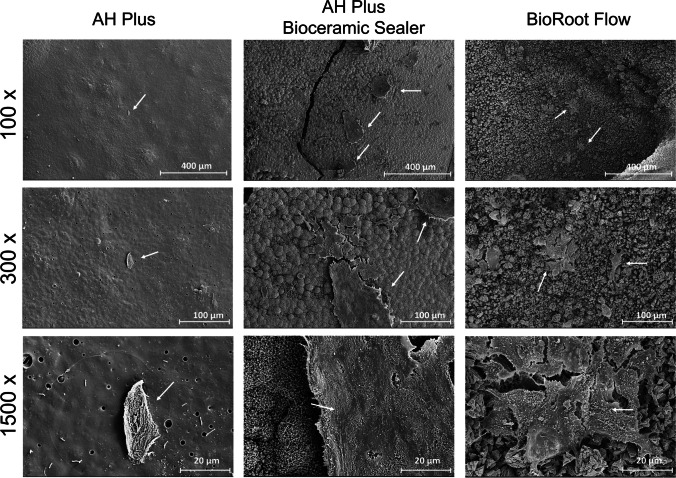
Results from SEM visualization after 72 h of culture of hPDLSCs seeded onto the surface of the tested sealer samples (BrF, AHPbcs, and AHP). Magnifications: 100X, 300X, and 1500X. Scale bars: 400 μm, 100 μm, and 20 μm

Representative results of the SEM visualization performed to qualitatively assess the morphology and adherence of hPDLSCs to the surface of the sealer samples are illustrated in Fig. 5. Both CSS samples exhibited a high number of functionally oriented elongated cells adhered to their surfaces. However, AHP samples showed a lack of adhered cells and debris, indicative of cellular death.

### Sealer bioactivity (RT-qPCR and Alizarin Red S staining)

**Fig. 6:**
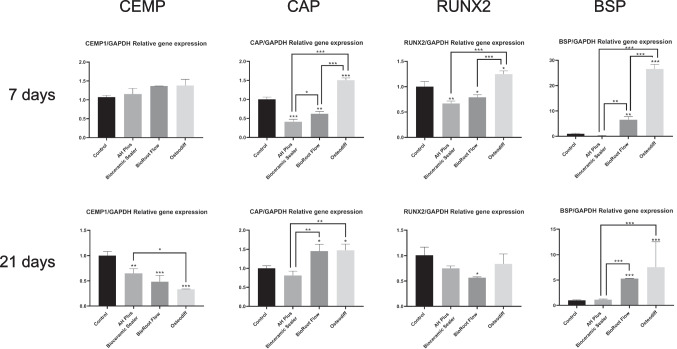
Results from the analysis of hPDLSCs osteo/odonto/cementogenic marker expression via RT-qPCR after 3, 7, 14 and 21 days of culture with the tested CSSs (BrF, AHPbcs), DMEM (negative control), or Osteodiff (postive control). *p < 0.05; **p < 0.01; ***p < 0.001. Two-way ANOVA analysis: asterisks above the bars indicate a significant difference with the negative control group; asterisks above the lines indicate a significant difference between the groups connected by the line

The results of the RT-qPCR performed to quantify hPDLSC osteo/cemento/odontogenic marker expression and assess the influence on cellular plasticity of the tested sealers are shown in Fig. 6. BrF-treated cells exhibited an overexpression of CAP and BSP after 21 days of culture compared to the negative control group (*p* < 0.05 and *p* < 0.001; respectively). AHPbcs-treated cells exhibited a similar expression of CAP, Runx2, and BSP after 21 days of culture compared to the negative control group. CEMP1 expression of both groups was similar to that of the control groups at 7 days but significantly declined after 21 days of culture (*p* < 0.01 for AHPbcs; *p* < 0.001 for BrF).

**Fig. 7:**
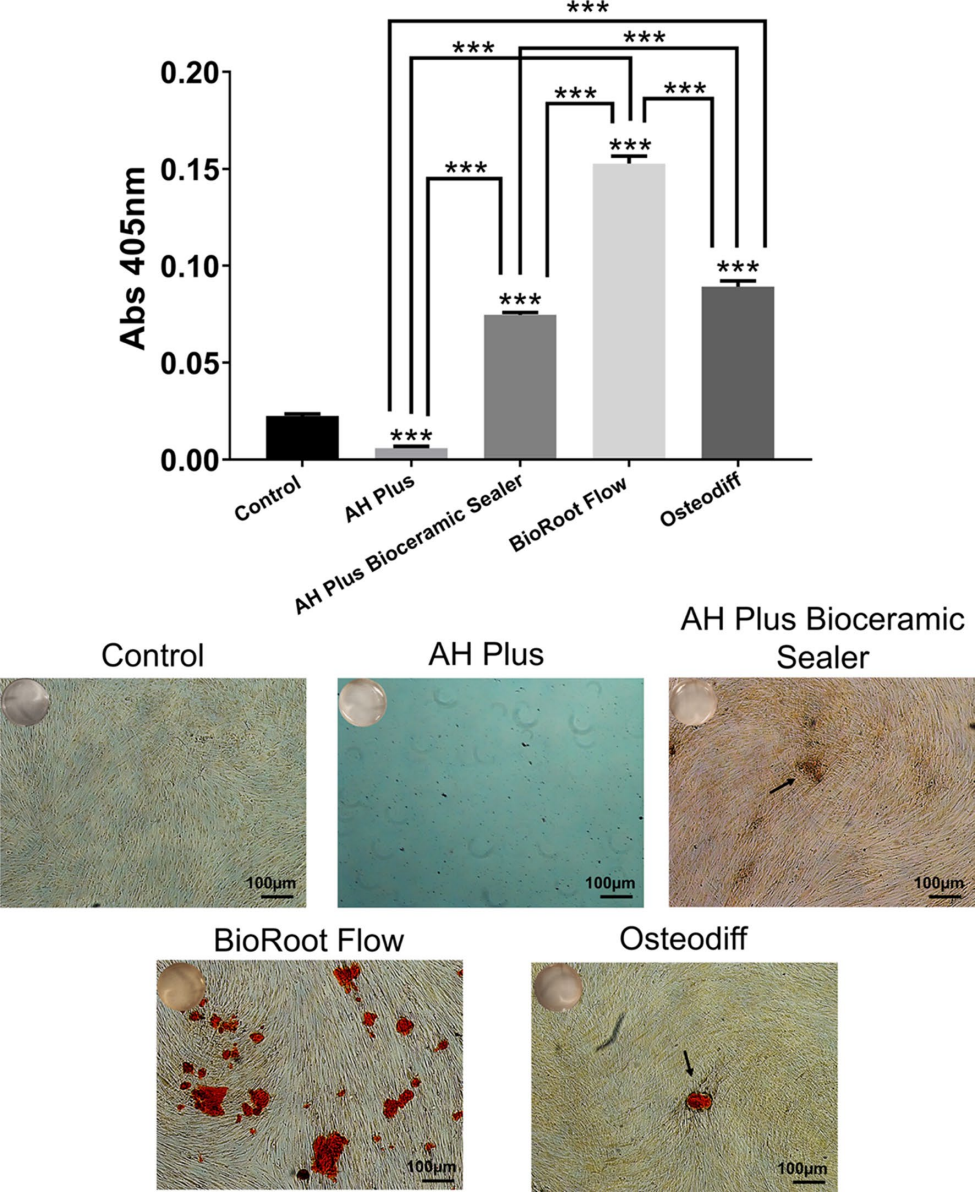
Results from the Alizarin Red S staining assay of hPDLSCs after 21 days of culture with the tested CSSs (BrF, AHPbcs), DMEM (negative control), or Osteodiff (postive control). *p < 0.05; **p < 0.01; ***p < 0.001. Two-way ANOVA analysis: asterisks above the bars indicate a significant difference with the negative control group; asterisks above the lines indicate a significant difference between the groups connected by the line. Representative images: red-stained areas indicate mineralization

The results and representative images of the Alizarin Red S staining performed to quantify hPDLSC calcified nodule formation and assess the bioactive potential of the tested sealers are shown in Fig. 7. AHP-treated cells exhibited a significantly lower mineralization than the negative and positive control groups (*p* < 0.001). AHPbcs-treated cells exhibited a significantly higher mineralization than the negative control group (*p* < 0.001), but significantly lower than the positive control group (*p* < 0.001). BrF-treated cells exhibited a significantly higher mineralization than the negative and positive control groups (*p* < 0.001). At the same time, the calcified nodule formation was significantly higher in the BrF-treated cells than the AHPbcs-treated cells (*p* < 0.001).

It should be highlighted that an inconsistency can be observed between the results of the Alizarin Red S staining assay and the RT-qPCR assay. BR-treated cells exhibit a significantly higher mineralization in the Alizarin Red S staining assay compared to the Osteodiff group (positive control), while the latter exhibited a significantly higher expression of differentiation markers. A possible explanation for this inconsistency is the high calcium content in Bioroot Flow, which may precipitate in the culture medium. Cells can easily accumulate this calcium irrespective of their differentiation, which may be higher in the Osteodiff medium. This highlights the importance of performing assays on bioactivity both from the perspective of cellular plasticity and mineralization potential.

### Sealer immunomodulatory properties (ELISA)

**Fig. 8:**
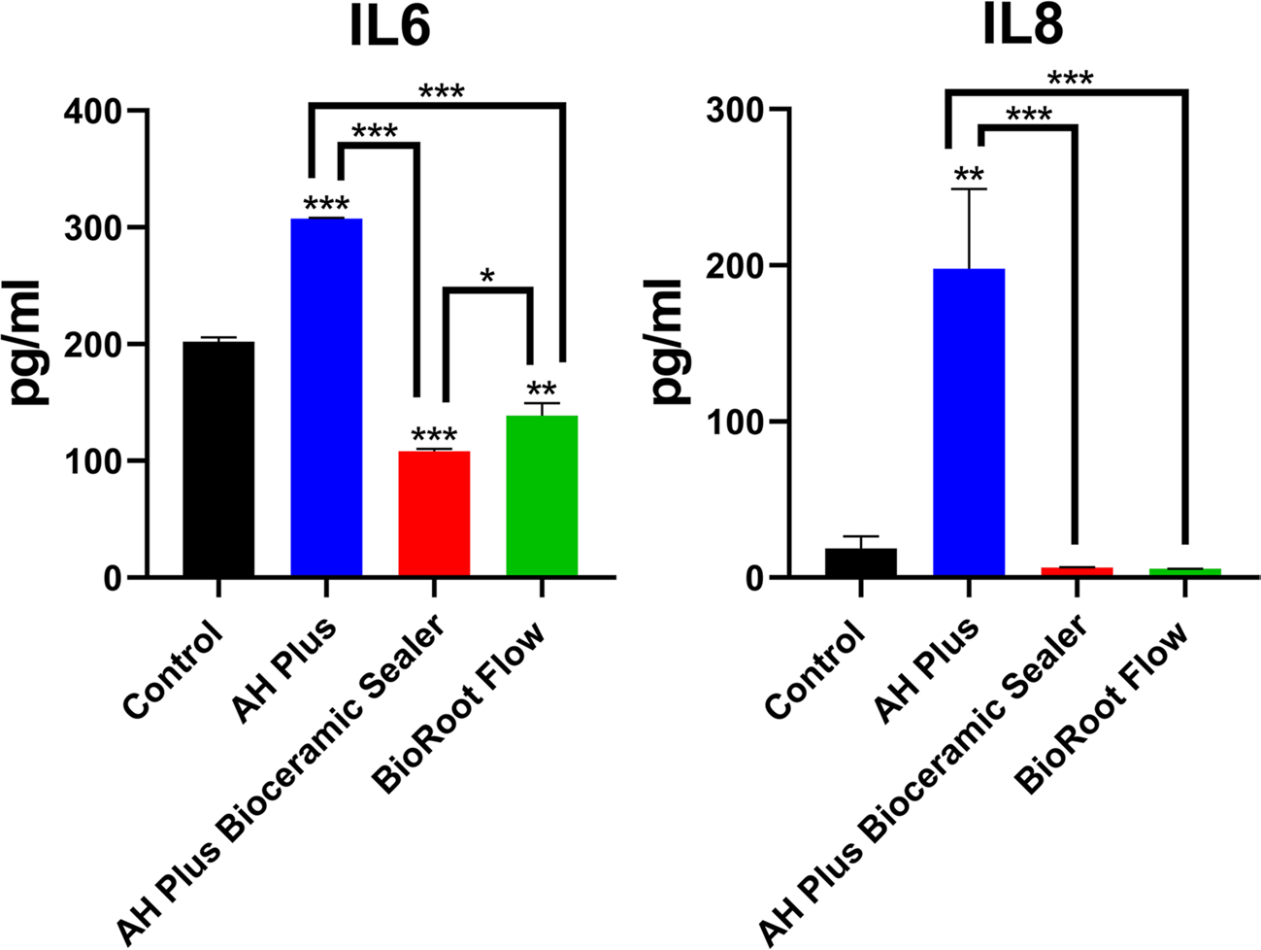
Results from the ELISA to assess hPDLSC expression of IL-6 and IL-8 after 72 h of culture with the testes sealers (BrF, AHPbs, and AHP) or in DMEM (negative control). *p < 0.05; **p < 0.01; ***p < 0.001. Two-way ANOVA analysis: asterisks above the bars indicate a significant difference with the negative control group; asterisks above the lines indicate a significant difference between the groups connected by the line

The results of the ELISA performed to quantify hPDLSC IL-6 and IL-8 expression and assess the immunomodulatory potential of the tested sealers are shown in Fig. 8. The expression levels of the pro-inflammatory cytokines IL-6 and IL-8 were significantly higher in AHP-treated cells than in the CSS-treated cells and in the control group. Both CSS-treated cells exhibited a significantly lower expression of IL-6 than the control group (*p* < 0.001 for AHPbcs; *p* < 0.01 for BrF) and a similar expression of IL-8.

## Discussion

Pre-mixed calcium silicate-based materials are becoming increasingly popular among clinicians and investigators. The “pre-mixed” format in a single syringe or capsule promises an easier handling and application, while presumptively maintaining their favourable biological properties [45]. The constant and rapid introduction of new CSSs hinders the production of scientific evidence to support or discourage their clinical use. For this reason, cellular studies appear as a relevant preliminary assessment to reliably confirm the biological safety of new materials and predict their behaviour in contact with living tissues [46]. Accordingly, the aim of the present in vitro study on hPDLSCs was to assess and compare the biological properties (cytocompatibility and bioactivity) and immunomodulatory potential of the pre-mixed CSSs BrF and AHPbcs.

The selection of the tested materials was based on the recent introduction of both CSSs, their shared clinical indications, and pre-mixed format. In fact, to the author’s knowledge, this is the first study on BrF and, to date, there is scarce evidence on the biological properties of AHPbcs and its immunomodulatory potential yet to be elucidated [25, 27, 30, 47]. Parallelly, AHP was used as a comparison, given its extensive evidence and common use as a control in similar studies [26, 48, 49].

**Fig. 9:**
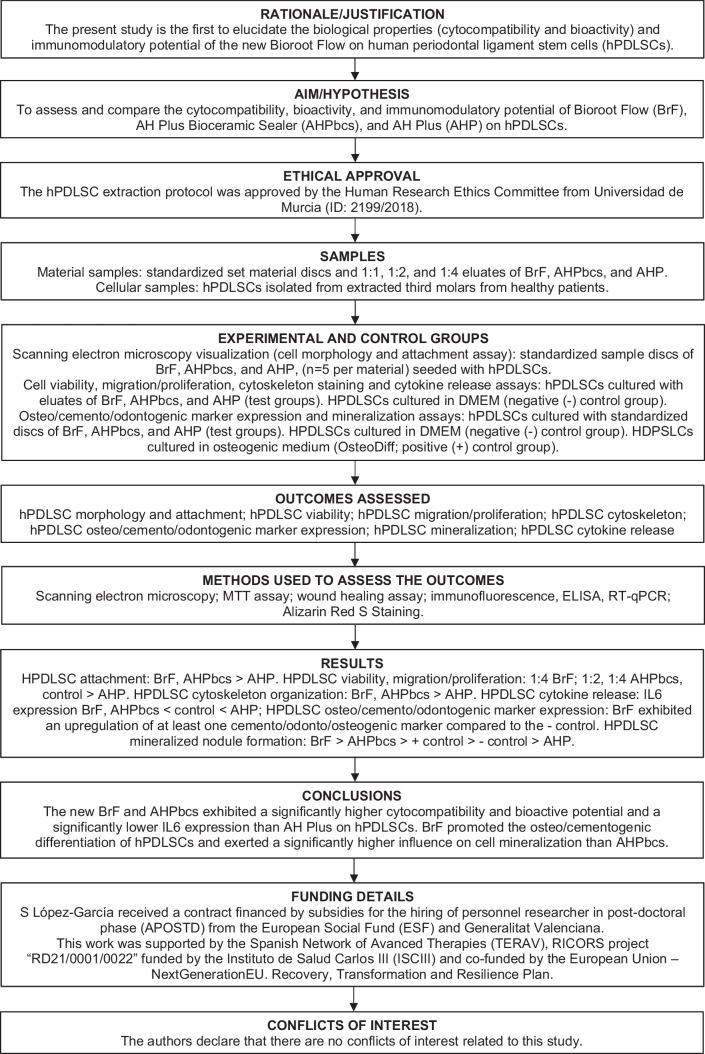
Preferred Reporting Items for Laboratory studies in Endodontology (PRILE 2021)-based flowchart [28]

The present study may act as preliminary evidence from which to develop studies on three-dimensional culture models, animal models, or clinical trials; as performed previously on other CSSs [3, 14, 50]. Currently, results should be interpreted considering they were obtained in controlled laboratory conditions, which do not take into account possible variables that could affect the sealer’s behaviour clinically [9, 51]. This acts as the main limitation of the present study. Nonetheless, in order to increase the reproducibility and transparency of this study, the recently introduced PRILE guidelines were followed throughout the manuscript [28]. Thus, the main steps of this work are illustrated in the PRILE flowchart (Fig. 9).

### Sealer cytocompatibility

Four different assays were performed to quantitatively and qualitatively assess the cytotoxicity/cytocompatibility of the tested sealers. Both quantitative measures, i.e., hPDLSC metabolic activity and migration, indicated a statistically significant cytocompatibility of BrF and AHPbcs and cytotoxicity of AHP, compared to a negative control group. The cytotoxicity of this epoxy resin-based sealers and other sealers and cements containing resin in their composition has already been reported [52, 53]. The negative cellular response towards AHP has been associated with the release of bisphenol-A and/or formaldehyde during its setting reaction [54, 55].

On the other hand, the favourable biocompatibility exhibited by the tested CSSs is consistent with previous evidence on other CSSs [56–58]. Specifically, AHPbcs also showed a favourable cytocompatibility on human periodontal ligament fibroblasts (hPDLFs) using an MTT [25] and an XTT assay [27] on previous studies.

Both qualitative analyses (SEM and immunofluorescence) complemented the results of the quantitative measures. hPDLSCs cultured together with BrF and AHPbcs showed an adequate morphology and attachment to the set sealers’ surfaces. The opposite occurred in AHP samples. The same was observed in previous similar studies on other CSSs [40, 59, 60]. Consistent with previous qualitative measures, the higher the dilution of the tested material, the greater the number of functionally oriented stained cells [34, 59].

Complementarily, in a previous study carried out by our research group, the cytocompatibility of AHPbcs was assessed on hPDLSCs and compared to that of Endosequence BC Sealer (ESbcs; Innovative Bioceramix, Vancouver, Canada) and AHP [30]. Similar standardized methods were used to assess the cytocompatibility of the tested materials (MTT assay, wound healing assay and SEM visualization), and the same tendency was observed: both pre-mixed CSSs exhibited an adequate cytocompatibility. The main components of BrF (tricalcium silicate and zirconium oxide) are shared with AHPbcs and ESbcs; and have been shown to be biocompatible in previous studies [61].

### Sealer bioactivity

Two different assays were performed to quantitatively assess the bioactivity of the tested sealers, one from the perspective of the material’s influence on cellular plasticity (RT-qPCR) and the other from the perspective of the material’s ability to promote mineralization (ARS).

From a cellular perspective, BrF-treated cells exhibited an overexpression of the cementogenic marker CAP and the osteogenic marker BSP. CAP acts as an indicator of periodontium repair and regeneration, since its overexpression is observed during the formation of cement, specifically during cell recruitment and differentiation [62, 63]. Parallelly, BSP acts as a tissue-specific indicator of mineralization, specifically expressed during the initial stages of osseous tissue formation [64–66]. Altogether, these results reflect the positive influence of BrF on the differentiation of hPDLSCs into a cemento/osteogenic lineage and its potential enhancement of periodontal/osseous tissue repair or regeneration.

Interestingly, no marker overexpression was observed from AHPbcs-treated cells. This contrasts with our previous report on AHPbcs, were an overexpression of CEMP1, CAP, RUNX2 and BSP markers was observed after 21 days of culture with hPDLSCs [30]. To date, there is no further evidence on the influence of AHPbcs on cellular plasticity. Therefore, the present results should be further confirmed and contrasted.

Regarding the sealers’ ability to promote mineralization, both BrF and AHPbcs-treated hPDSCs exhibited a significantly higher mineralization production compared to the negative control group. This coincides with our previous report on AHPbcs [30]. However, differing from said report, AHPbcs-treated cells evidenced a significantly lower calcified nodule formation compared to the positive control group. In fact, BrF-treated cells produced significantly superior results in the ARS assay compared to AHPbcs and the positive control group. Altogether, these results reflect both material’s ability to promote mineralization and suggest that BrF may present a greater potential than AHPbcs in this regard.

Nevertheless, a recent study on the bioactivity of various CSSs from a physicochemical perspective, i.e., apatite forming ability, adds evidence on AHPbcs’s ability to form a mineral layer on its surface. Specifically, calcium phosphate and calcium carbonate was detected by means of micro-Raman [47].

### Sealer immunomodulatory potential

ELISA assay revealed that AHP-treated cells exhibited a significant overexpression of the IL-6 and IL-8 compared to the control group and the tested CSSs. Root canal sealers, as a foreign body, react with periapical tissues and commonly upregulate inflammatory cytokines such as IL-6, IL-8, IL-12, and TNF-α in early stages of inflammation, which results in an increased cytotoxicity to local cells and in turn hinders tissue repair and regeneration [67–69].

ELISA assay also revealed that both CSS-treated cells exhibited a significant underexpression of the IL-6 and a similar expression of IL-8 to that of the control group. Recently, several studies reported CSSs may have immunomodulatory effects on inflammation and osteogenesis [70, 71]. This immunomodulatory potential follows various mechanisms, such as the regulation of cytokines release and the influence on macrophage phenotypes [20]. Thus, the under-regulation of IL-6 showed by BrF and AHPbcs can act as an indicator of their immunoregulatory potential.

## Conclusion

The new calcium silicate-based sealers Bioroot Flow and AH Plus Bioceramic Sealer exhibit adequate and comparable cytocompatibility on hPDLSCs. Bioroot Flow also promoted the osteo/cementogenic differentiation of hPDLSCs. Both calcium silicate-based sealers favored the downregulation of the inflammatory cytokine IL-6 and the calcified nodule formation from hPDLSCs. Bioroot Flow exerted a significantly higher influence on cell mineralization than AH Plus Bioceramic Sealer.

### Supplementary information

Below is the link to the electronic supplementary material.

Supplementary file1 (DOCX 27 KB)

## Data Availability

No datasets were generated or analysed during the current study.
